# BindPred: a framework for predicting protein–protein binding affinity from language model embeddings

**DOI:** 10.1093/bioinformatics/btag309

**Published:** 2026-05-14

**Authors:** Haixing Piao, Veda Sheersh Boorla, Somtirtha Santra, Costas D Maranas

**Affiliations:** Department of Chemical Engineering, The Pennsylvania State University, University Park, PA 16802, United States; Department of Chemical Engineering, The Pennsylvania State University, University Park, PA 16802, United States; Department of Chemical Engineering, The Pennsylvania State University, University Park, PA 16802, United States; The Center for Bioenergy Innovation, Oak Ridge National Laboratory, Oak Ridge, TN 37831, United States; Department of Chemical Engineering, The Pennsylvania State University, University Park, PA 16802, United States; The Center for Bioenergy Innovation, Oak Ridge National Laboratory, Oak Ridge, TN 37831, United States

## Abstract

**Motivation:**

Reliable predictions of protein–protein binding affinities are essential for molecular biology and therapeutic discovery. However, most computational methods rely on three-dimensional structural models, which are often unavailable for many complexes.

**Results:**

We introduce BindPred, a structure-agnostic input framework that predicts affinities directly from amino acid sequences by combining embeddings from large protein language models with gradient boosting trees. On the protein–protein binding (PPB)-Affinity benchmark, which comprises 11 919 diverse complexes, BindPred achieves a Pearson correlation coefficient of 0.86 in random split five-fold cross-validation. Ablation analysis indicates that evolutionary embeddings alone capture most of the predictive signals, while augmenting with physics-based energy terms from PyRosetta and BindCraft increases the correlation only by 0.01. A more stringent protein-level split that places entire protein families (wild-type and all mutants) exclusively in either training or testing sets, resulting in only a modest decline in performance, demonstrating robust generalization to novel interaction pairs. Because BindPred operates exclusively on sequence input, it enables rapid inference [approximately 3 million complexes per GPU (T4) hour], making proteome-scale screening computationally feasible.

**Availability:**

The pretrained model and inference pipeline are available in a Google Colab notebook: BindPred Colab notebook. The training dataset, code, and model weights are available on the hugging face: https://huggingface.co/hbp5181/BindPred.

## 1 Introduction

Protein–protein interactions (PPIs) govern essential cellular processes from immune recognition to metabolic regulation, making accurate binding affinity prediction ($K_{d}$ or ${\Delta G}_{\mathrm{bind}}$) indispensable for both fundamental biology and drug discovery (Sledzieski *et al.* 2021). Experimental techniques such as surface plasmon resonance (Sparks *et al.* 2019) and isothermal titration calorimetry (Csibra and Stan 2022) provide precise measurements but require purified proteins and specialized equipment, limiting throughput to hundreds rather than the millions of interactions needed for proteome-wide studies.

To overcome experimental throughput limitations, multiple computational strategies have been developed. Physics-based methods, including molecular dynamics (MD), free-energy perturbation (FEP), and thermodynamic integration, estimate ${\Delta G}_{\mathrm{bind}}$ by decomposing it into enthalpic and entropic contributions from electrostatics, hydrogen bonding, van der Waals forces, and conformational entropy (Singh and Warshel 2010, Kříž *et al.* 2023). These mechanistically rigorous methods require several demanding inputs: high-quality starting structures, carefully tuned force fields, and extensive simulation trajectories to achieve convergence. Even under optimized protocols, calculations often take hours per complex (Liu *et al.* 2024). Due to high computational cost of physics-based simulations, surrogate machine learning methods have also been explored. Structure-based machine learning (Chaves *et al.* 2025, Lebedenko *et al.* 2025, Thomas *et al.* 2025) accelerates prediction by learning features derived from protein complexes yet remains bottlenecked by structure generation. Even advanced structure predictors such as AlphaFold2 (Jumper *et al.* 2021), AlphaFold-Multimer (Evans *et al.* 2021), and AlphaFold3 (Abramson *et al.* 2024) frequently misplace binding partners, produce overlapping chains, or distort binding interfaces, especially for multichain assemblies with weak or absent coevolutionary signals (Bryant *et al.* 2022, Wee and Wei 2024). These limitations have motivated the development of approaches that bypass structure prediction entirely.

Protein language models (pLMs) based models address this challenge by extracting structural and functional information directly from sequences (Elnaggar 2021). Trained on hundreds of millions of sequences, models such as the Evolutionary Scale Model 2 (ESM2) (Lin *et al.* 2023) learn context-aware embeddings that capture evolutionary constraints, local chemistry, and functional motifs without requiring explicit structural input. Multimeric Interaction Transformer (MINT) (Ullanat *et al.* 2026) builds on the 650M-parameter ESM-2 backbone by integrating cross-chain attention blocks and adapting the masked language modeling objective for multi-sequence input, enabling the model to learn contextual representations of interacting protein sets. These embeddings enable structure-free inference in a single forward pass (Lam *et al.* 2024) and have proven effective for function annotation and mutation effect prediction (Marquet *et al.* 2022).

We present BindPred, a structure-free binding affinity predictor integrating pLM embeddings with gradient boosting trees (GBT). Operating directly on sequences, BindPred avoids errors from docking or homology modeling and applies to complexes lacking reliable structures. The embeddings, derived from ESM2 or MINT, remain fixed during training and serve as task-agnostic biochemical descriptors. With only ∼32 000 trainable parameters (11 919 dataset with 2560 input features), the architecture operates on precomputed embeddings and can evaluate approximately three million complexes per GPU (T4) hour, achieving speeds that are orders of magnitude faster than FEP protocols or structure-based machine learning pipelines.

Several models have incorporated pLM embeddings into protein–protein interaction predictors, but most focus on relative ΔG prediction (Gurusinghe *et al.* 2025), or interface-specific architectures (Sargsyan and Lim 2024, Banerjee *et al.* 2025). ProBASS (Gurusinghe *et al.* 2025) exemplifies the latter by combining ESM2 embeddings with structure-conditioned vectors from ESM-IF1 (Hsu *et al.* 2022) to predict ΔΔG upon mutation. Although effective on SKEMPI-style datasets, ProBASS requires high-confidence structural models for each variant and predicts relative changes in affinity, making it unsuitable for ranking entirely novel complexes. In contrast, BindPred directly estimates absolute $\log_{10}K_{d}$ values from amino acid sequences, eliminating structural dependencies, enabling high-throughput screening, and maintaining strong predictive accuracy across diverse protein interactions.

We evaluated BindPred against a sequence–structure hybrid baseline on the PPB Affinity benchmark (Liu *et al.* 2024), which spans antibody–antigen, enzyme–inhibitor, and receptor–ligand complexes. Using random split five-fold cross-validation and strict protein-level splits, we assessed predictive accuracy and generalizability. We also trained variants of BindPred that incorporated structural features from PyRosetta (Chaudhury *et al.* 2010) and BindCraft (Pacesa *et al.* 2025) to quantify their added value. Across all settings, the embedding-only model matched the augmented variants, achieving a Pearson correlation coefficient of 0.86 under stratified splitting and maintaining strong performance on entirely unseen proteins. Because it operates directly on sequence input, BindPred can rapidly screen millions of protein pairs, enabling proteome-wide affinity prediction and quantitative ranking of *de novo* designed sequences, including binders generated by structure-conditioned inverse-folding methods such as ProteinMPNN (Dauparas *et al.* 2022).

## 2 Methods

### 2.1 Overview of the BindPred framework

**Figure 1:**
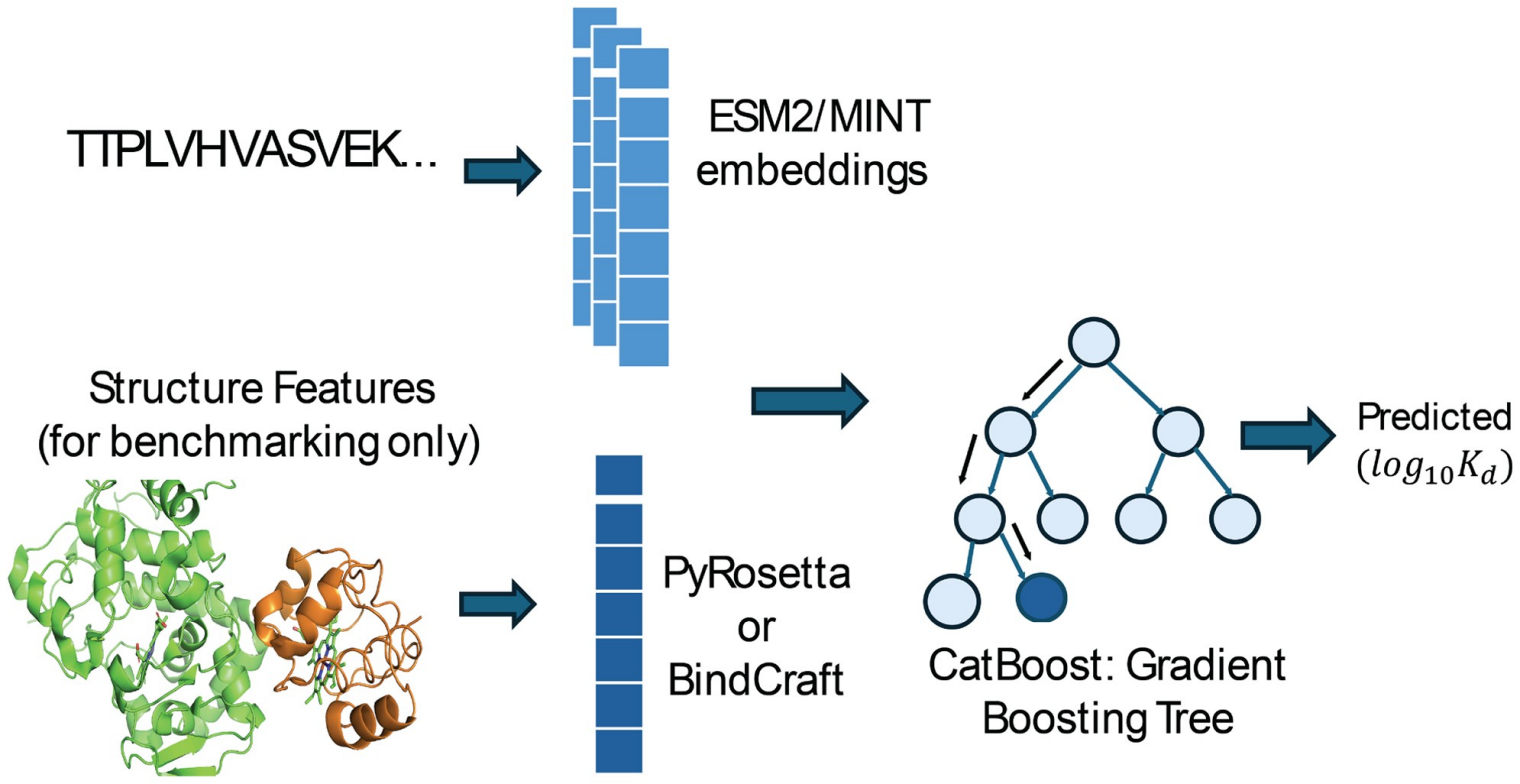
BindPred model architecture and workflow. Protein sequences are encoded with ESM2 or MINT embeddings and used to train a CatBoost regressor to predict $\log_{10}K_{d}$. Structural energy terms are optionally appended for benchmarking but excluded from the default model. The default model in the Google Colab notebook only requires ESM2 embedding inputs.

Figure 1 provides an overview of the training and inference procedures employed in the BindPred framework.

### 2.2 Dataset and feature extraction

#### 2.2.1 “Corpus PPB-Affinity” dataset

We refer to Corpus PPB-Affinity as the combined dataset of 11 919 protein–protein complexes drawn from SKEMPI v2.0, SAbDab, PDBbind 2020, Affinity Benchmark v5.5, and ATLAS, and distinguish it from the structure-dependent PPB-Affinity baseline model (Liu *et al.* 2024). For BindPred models (Figure 1) that combined embeddings with physical energy terms, we used 9597 complexes with PyRosetta (19 energy features) and 3468 complexes with BindCraft (14 energy features), the latter reduced due to segmentation errors.

#### 2.2.2 Sequence feature extractions

Sequence-level embeddings were obtained by mean pooling the residue-level hidden states from the final transformer layer of either the ESM-2 (esm2_t33_650M_UR50D; Lin *et al.* 2023) model, pretrained on UniRef50 with 650 M parameters, or MINT (Ullanat *et al.* 2026) (see Supplementary Information for more details). These features were extracted separately for each binding partner and concatenated by joining two 1280 features to form a single 2560-dimensional representation for one affinity value.

#### 2.2.3 Structural feature extractions

Structural conformation and stability features were computed using PyRosetta (Chaudhury *et al.* 2010). All input structures were first relaxed with the FastRelax protocol under the Rosetta ref2015 all-atom energy function to remove steric clashes and optimize side-chain packing. Each relaxed structure was then rescored in PyRosetta 4 (release 2024.12) using default ref2015 weights with the *flags ignore_unrecognized_res true, ex1*, and *ex2aro* to enable thorough side-chain sampling. For every wild-type and mutant pose, we extracted the full set of nineteen ref2015 all-atom energy terms, recording both the raw energy values and fourteen derived interaction-energy features. The ref2015 scoring framework is described in detail by Alford *et al.* (2017), and the interaction-energy calculation protocol follows the BindCraft methodology (Pacesa *et al.* 2025). The BindCraft energy calculations fail when there are multichain (e.g. two receptor chains with one ligand chain or vice versa), so only two-chained complexes were used for the BindCraft energy calculations.

### 2.3 Cross-validation split strategies and model training

Corpus PPB-Affinity datasets were randomly split (random seed = 42) into training (80%) and testing (20%) sets to optimize the model weights on a subset of data while evaluating its performance on unseen examples for the fivefold cross validations ((Figs. 3, 5, 6, and 7). The training script is available in train.py.

Protein-level splitting (Fig. 7) was applied to prevent information leakage by grouping each wild-type complex with all its variants under the same PDB_ID, ensuring that no wild-type–mutation pairs crossed the train–test boundary. Model performance was assessed using five-fold group-aware cross-validation, maintaining an overall 80% training and 20% testing ratio. In each fold, 20% served as the test set and the remaining 80% as the training set.

BindPred was trained using the CatBoost gradient boosting framework with root mean square error (RMSE) as the objective function. Detailed hyperparameters and feature variants are provided in Supplementary Methods.

## 3 Results

We evaluated BindPred across multiple benchmarking scenarios to assess its predictive accuracy and generalization capability. We began by comparing sequence-only models based on ESM2 and MINT embeddings to a hybrid baseline on the comprehensive Corpus PPB-Affinity dataset and its individual subsets. We then conducted cross-dataset validation to evaluate robustness under distribution shifts, followed by a stringent protein-level data split, defined by PDB ID, to assess model performance on entirely unseen proteins. Finally, we explored the applicability of BindPred in ranking *de novo* protein designs, providing a comprehensive assessment of its accuracy and scalability across diverse prediction settings.

### 3.1 Data structure

A total of 11 919 protein complexes were extracted from the Corpus PPB-Affinity dataset aggregated from five different sources. These complexes span $\mathrm{lo}g_{10}K_{d}$ values from −15.7 to −1.3, with 74.9% of the data falling between −10 to −5. Table S1 and Fig. S1 in the supplementary information provide an overview of the overall dataset.

### 3.2 Fivefold cross-validation on the corpus PPB-Affinity

BindPred was evaluated on the full Corpus PPB-Affinity (12 062 total with 11 919 valid complexes) using two protein embedding approaches, ESM2 and MINT, and compared against the PPB-Affinity baseline model. ESM2 provides general-purpose protein embeddings trained on large-scale protein sequence data. MINT extends the 650M-parameter ESM2 architecture by incorporating cross-chain attention mechanisms and undergoes additional training on a curated PPI dataset derived from STRING (Szklarczyk *et al.* 2024), which enables explicit modeling of interchain residue relationships. In BindPred, embeddings were derived from full-length sequences, with both interacting proteins input simultaneously. Self-attention operates within each protein chain while cross-attention enables information exchange between chains. The resulting protein-specific embeddings were then extracted and concatenated to form a 2560-dimensional representation for downstream prediction.

**Figure 2:**
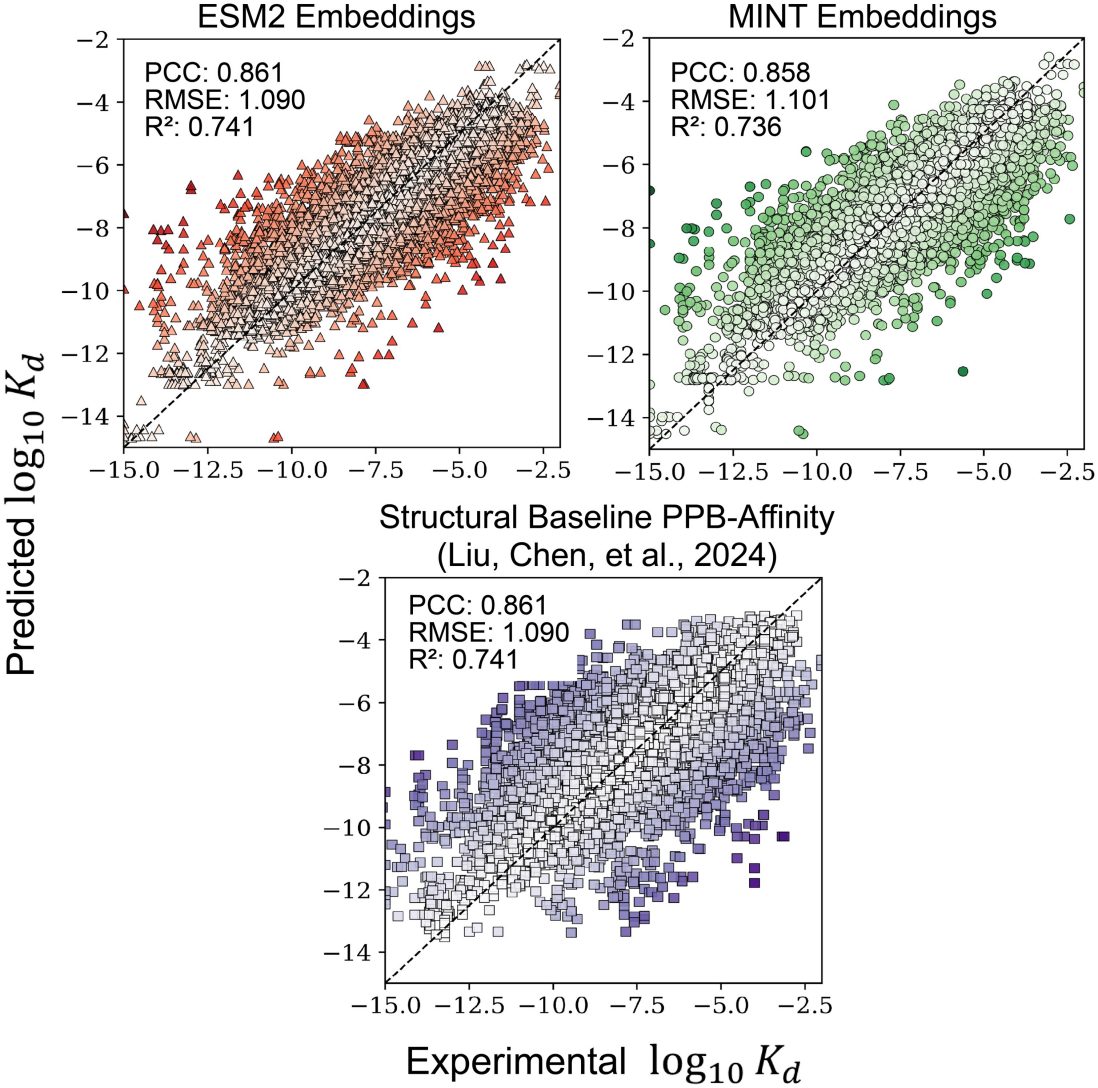
Sequence-only BindPred outperforms the Structural Baseline PPB-Affinity model in random split fivefold cross-validation. Predicted $\log_{10}K_{d}$versus experimental values for 11 919 protein–protein complexes under random split five-fold cross-validation. Points are colored by absolute error, with closer alignment to the diagonal indicating higher predictive accuracy. BindPred with ESM2 embeddings and with MINT embeddings both outperform the baseline PPB-Affinity model.

Under Random Split Five-Fold Cross-Validation in Fig. 2, both embedding-based BindPred models exceeded the Structural Baseline PPB-Affinity’s PCC by approximately 0.16. Paired t-tests on per sample absolute prediction errors computed from out of fold predictions confirmed the improvements were statistically significant for both ESM2 (ESM2 :  $P\;=\;2.3\;\times \;10^{-10})\;$and MINT ($P\;<\;10^{-10}$). In addition, both ESM2 and MINT embedding based models performed nearly identically with a PCC difference of 0.003. While a paired t-test on absolute prediction error confirms the result was statistically significant ($P\;<\;10^{-10}$), the magnitude of difference was negligible.

These performance differences primarily arise from fundamental differences in feature representation and scaling between the two approaches. The Structural Baseline PPB-Affinity crops each complex to 128 interface residues and encodes all pairwise geometry relationships between interface residues, resulting in 128^2^ = 16 384 features. The quadratic scaling increases computational cost and amplifies sensitivity to coordinate noise as perturbations in a single amino acid propagate across many pairwise features. In contrast, embedding-based BindPred models use fixed-length, sequence-derived representations that do not depend on explicit structural coordinates, thereby reducing sensitivity to structural noise and enabling more robust generalization across diverse complexes.

Despite the overall advantage of the embedding-based models, they exhibit three limitations. First, extreme affinity outliers are present. For example, the Colicin E2 and Im2 complex (PDB 3U43, experimental $\log_{10}K_{d}$ = −14.4) was overestimated by ESM2 with a value of −6.2 and MINT at −6.9, with only partial correction from the structural baseline at −7.7. In addition, an engineered TNF alpha affibody (PDB 2TGP, experimental −6.0) was underpredicted by both sequence-only BindPred models at −12.6. Both cases involve deeply buried interfaces near 2200 Å^2^ and dense salt bridge networks that are not explicitly encoded in sequence embeddings and are only coarsely captured by baseline descriptors. Second, for certain mutations, the embeddings show limited sensitivity because both ESM2 and MINT primarily encode evolutionary statistics from the sequence. When a substitution carries a weak or ambiguous evolutionary signal in the alignment, the models infer little effect and predict that the mutant will behave exactly as the wild type. Third, not surprisingly, performance declines for underrepresented complex families with few close analogs in training.

### 3.3 Fivefold cross-validation on individual subset

As shown in Fig. S1, the Corpus PPB-Affinity dataset comprises five subsets originating from different source databases, each reflecting distinct experimental conditions, complex types, and structural quality criteria. Evaluating BindPred and the Structural Baseline PPB-Affinity model on each subset separately enables us to assess how well the models generalize across distinct data sources and to identify performance trends that differences in interface size, sequence diversity, or experimental resolution may drive.

The subset level analysis helps to pinpoint subset-specific challenges that may not be apparent in the aggregated results. Table 1 summarizes average performance of embedding based models for each subset, where the model was trained and evaluated on the same subset. The Affinity Benchmark v5.5 subset yielded the highest overall performance for both ESM2 and MINT embedding of BindPred. Although BindPred is a sequence-only model, this dataset consists of well-characterized protein complexes with high-quality experimental affinity measurements (Vreven *et al.* 2015), providing a more consistent relationship between sequence-derived features and binding strength and supporting more accurate affinity ranking and regression.

**Table 1:** Five-fold cross-validation average results for each individual subset.

Subset	PCC			RMSE		
	ESM2	MINT	PPB	ESM2	MINT	PPB
Benchmark	0.895	0.886	0.671	1.011	1.052	1.618
ATLAS	0.632	0.657	0.387	0.963	0.934	1.169
PDBbind	0.824	0.820	0.662	1.175	1.188	1.627
SAbDab	0.661	0.666	0.335	0.954	0.948	1.447
SKEMPI	0.898	0.891	0.745	0.891	0.916	1.544

Benchmark, affinity benchmark v5.5; PPB, structural baseline PPB-affinity.

The SKEMPI v2.0 dataset also exhibits strong correlation performance with BindPred (i.e. PCC = 0.89). The SKEMPI v2.0 dataset is dominated by mutations introduced on a fixed wild-type scaffold, with approximately three-quarters of entries involving a single amino acid substitution and most remaining cases consisting of double substitutions (Jankauskaite *et al.* 2019, Liu *et al.* 2024). This controlled mutational regime limits sequence variability while preserving a shared structural and evolutionary context across variants. Such conditions favor embedding-based models, which encode contextual residue dependencies and can resolve fine-grained sequence perturbations within a stable background, thereby enabling accurate mapping between localized sequence changes and binding affinity differences.

In contrast, the ATLAS subset posed the greatest challenge for BindPred across both ESM2 and MINT embeddings. ATLAS primarily comprises TCR-pMHC interactions derived from the original ATLAS database (Borrman *et al.* 2017), representing a biologically specialized and structurally constrained interaction class. These datasets often exhibit limited evolutionary diversity and shallow alignment depth, reducing the amount of transferable coevolutionary signal available to pretrained language model embeddings. Consequently, predictive performance declines relative to more heterogeneous protein–protein interaction benchmarks, where broader evolutionary sampling supports more robust sequence-derived representations.

### 3.4 Cross-subset validation

Each subset differs substantially in biological interaction scope and experimental data generation, resulting in distinct statistical and structural properties across datasets. Affinity Benchmark v5.5, for example, compiles binding affinities primarily from high-quality biophysical measurements, including surface plasmon resonance (SPR) and isothermal titration calorimetry (ITC), with values verified against original publications and converted to binding free energies where necessary (see Table S1 for detailed subset information in the Supplementary).

By training on four subsets and evaluating on the held out fifth (“stranger” validation). Model robustness was assessed under controlled distribution shifts induced by differences in interaction class, experimental measurement methodology, assay precision, and curation practices.

**Figure 3:**
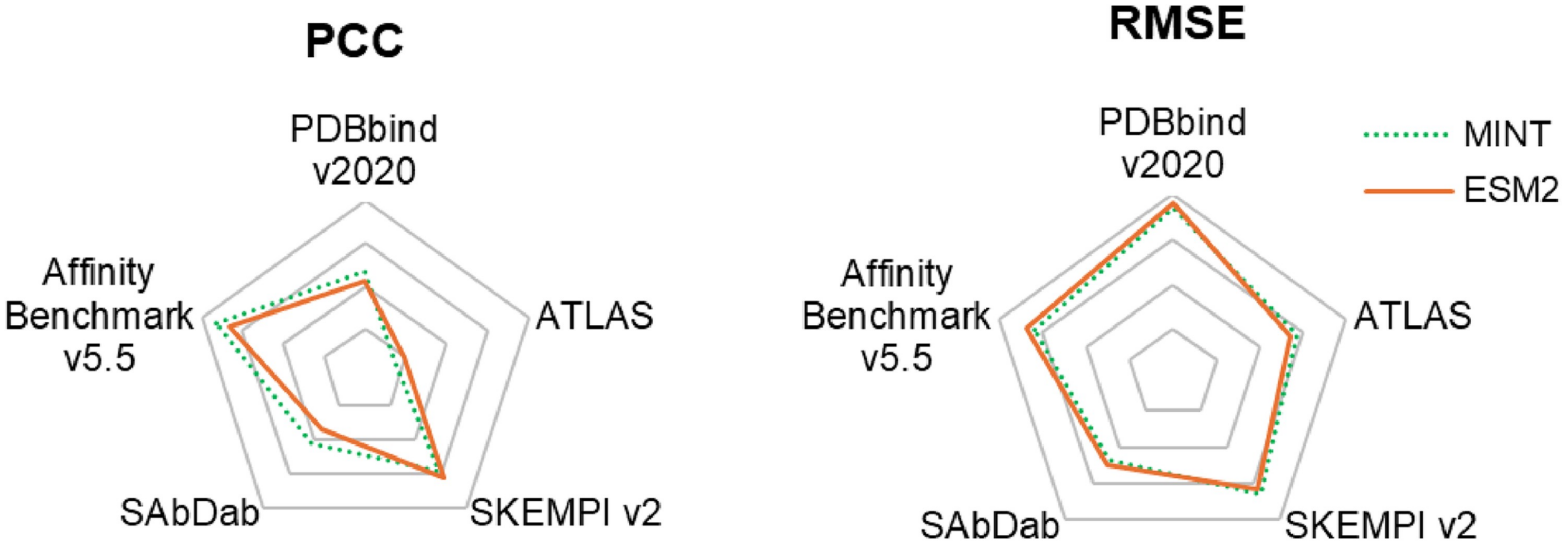
Cross-subset “Stranger” validation. Radar plots show the performance of MINT-based (green, dashed) and ESM2-based (red, solid) BindPred models when trained on four datasets and tested on the fifth. Performance varies substantially by held-out dataset, with Affinity Benchmark v5.5 yielding the highest cross-dataset accuracy and ATLAS showing the largest performance drop. Differences between MINT and ESM2 are modest but consistent, with MINT achieving higher PCC and lower RMSE, suggesting better resilience to domain shifts, although neither model fully overcomes the challenges posed by heterogeneous or structurally distinct interaction types.

Under cross-subset stranger validation, both versions of BindPred retained measurable predictive signal despite complete separation of interaction classes between training and testing sets. PCC values in this setting are lower than those observed in subset-specific fivefold cross-validation (Fig. 3, Table 2), indicating reduced performance under distribution shift. However, both ESM2 and MINT embeddings retain predictive signal. MINT embeddings achieve higher PCC in PDBbind v2020, SAbDab, and the Affinity Benchmark v5.5 set, whereas ESM2 embeddings perform better in ATLAS and SKEMPI datasets.

**Table 2:** Cross-subset stranger validation metrics.

Subset	PCC		RMSE	
	ESM2	MINT	ESM2	MINT
PDBbind v2020	0.423	0.472	1.902	1.846
ATLAS	0.194	0.147	1.351	1.423
SKEMPI v2	0.629	0.587	1.576	1.638
SAbDab	0.346	0.427	1.236	1.183
Benchmark	0.667	0.727	1.693	1.594

Datasets such as PDBbind v2020 and SabDab contain complexes with experimentally resolved structures and well-defined binding interfaces, often involving conserved contact patterns across related complexes. These properties are consistent with interaction patterns represented in large-scale interaction databases such as STRING (Szklarczyk *et al.* 2024), which compiles protein–protein associations inferred from functional evidence including coexpression, gene neighborhood proximity, text mining, and evolutionary co-occurrence. ATLAS integrates affinity measurements obtained from diverse assay types and experimental conditions and includes complexes with broader structural and interaction variability. This heterogeneity introduces greater variation between sequence-derived interaction patterns and experimentally determined binding determinants. SKEMPI v2 focuses on mutation-induced affinity changes and includes substitutions at both interfacial and non-interfacial residues, which reduces reliance on interaction-pair representations and increases the importance of full-sequence context captured by models such as ESM2.

### 3.5 Structure-based energy features from PyRosetta and BindCraft

Recognizing the limitations of sequence-only approaches, including performance saturation and reduced accuracy at extreme binding affinities, structural information was incorporated into the training dataset. ESM-2 embeddings were selected as the sequence representation due to their strong performance across diverse protein modeling tasks. To evaluate the contribution of physics-based structural features, PyRosetta energy terms were computed for 9597 protein complexes, capturing van der Waals interactions, solvation effects, and hydrogen bonding. In addition, BindCraft energy features were extracted for 3468 complexes using a fast relaxation protocol to quantify interface-specific energetics. These structural descriptors were then appended to the ESM-2 embeddings.

**Figure 4:**
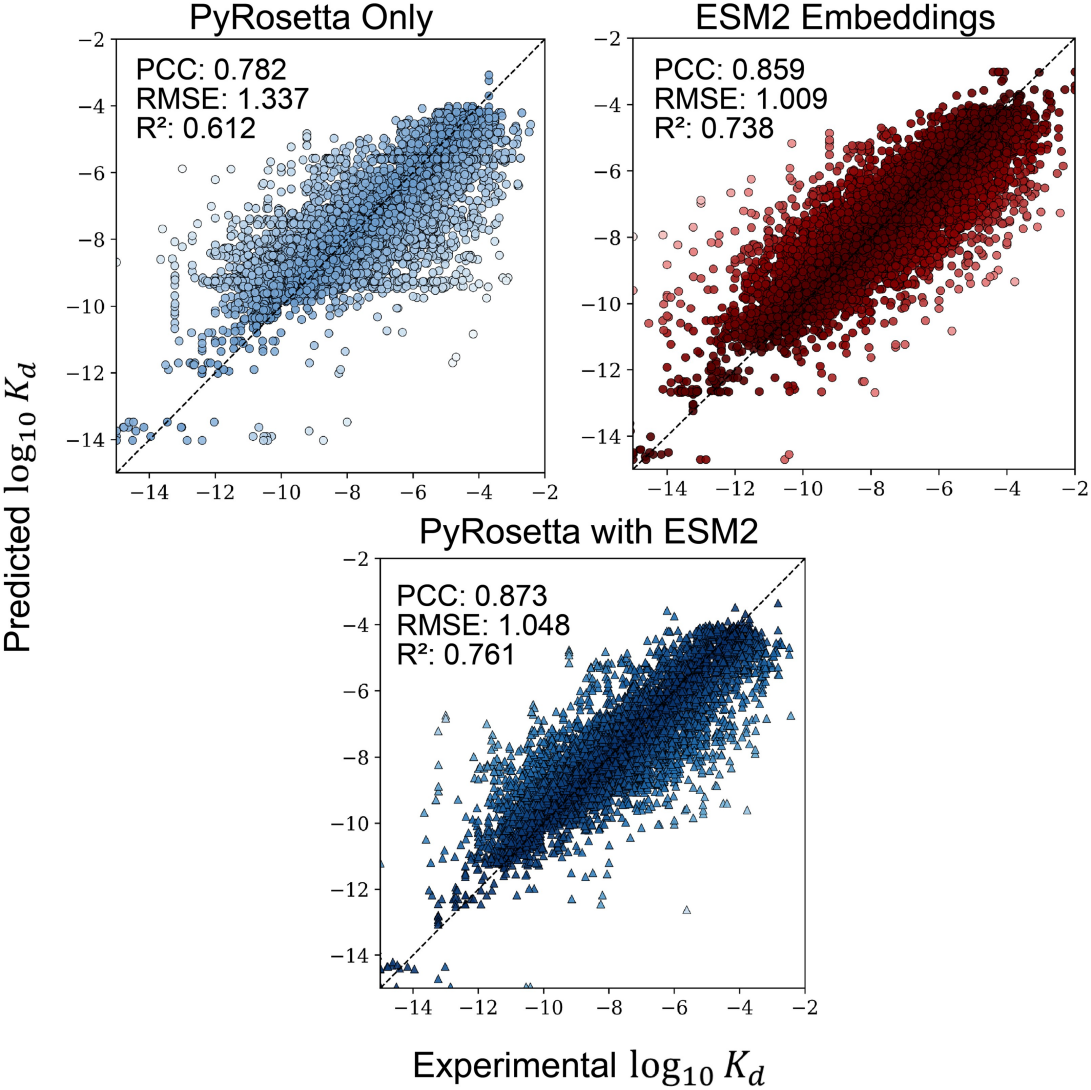
Random split fivefold cross validations across n = 9597 protein–protein complexes for three feature sets. PyRosetta Only uses a 19-dimensional vector of interface energy terms derived from PyRosetta. ESM2 embeddings use 2560 features obtained by mean pooling both protein embeddings and concatenating embeddings. PyRosetta with ESM2 combines both for a 2579-dimensional input. All three variations have been trained using the same protein complexes and hyperparameters indicated in the “Methods” section.

Figure 4 shows the performance difference between PyRosetta-only features and ESM-2 embeddings was substantial, with the embedding model showing higher correlation (PCC increased by 0.077 while RMSE decreased by 0.126). Nevertheless, PyRosetta features alone were able to describe binding affinity using only 20 physics-based energy terms, reflecting the strong contributions of structural packing, hydrogen bonding, solvation energetics, and other physical determinants of binding affinity. When these energy descriptors were combined with the 2560-dimensional embedding input (2579 total features), the resulting performance improvement was minimal.

**Figure 5:**
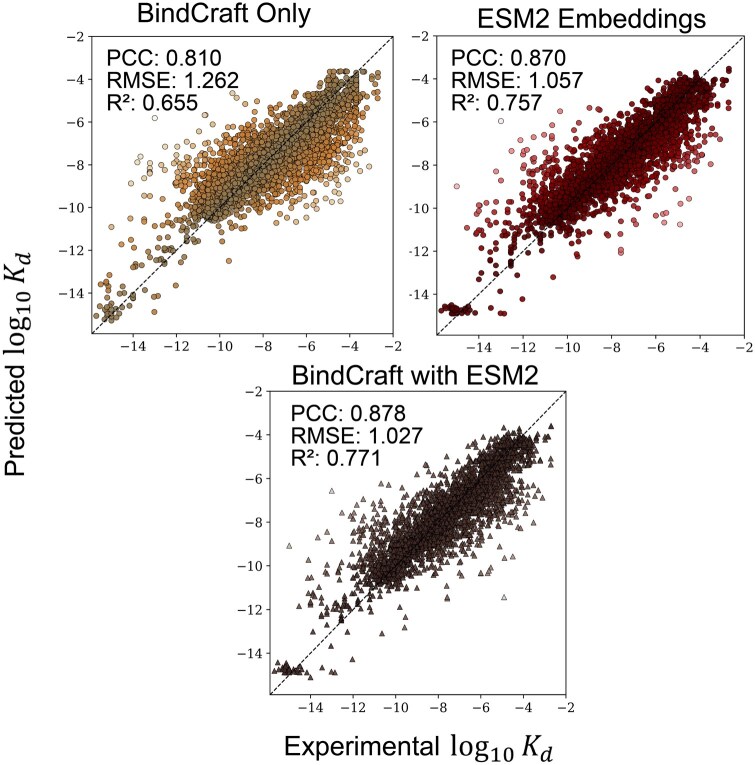
Random split fivefold cross validations across *n* = 3468 protein–protein complexes for three feature sets. Of 3468 complexes, nearly half of this subset came from SKEMPI v2 entries, which are enriched in point mutations, while the remaining complexes were drawn from four other sources with more heterogeneous interaction types. BindCraft frequently failed for multichain assemblies (e.g. two receptor chains with one ligand or vice versa), so this subset should be viewed as a biased sample rather than a representative slice of the full dataset.

Figure 5 compares the performance of three models: BindCraft-only features, ESM-2 embeddings, and a combined ESM-2 plus BindCraft feature model. When the embedding-only model was retrained on the subset of complexes for which BindCraft energies were successfully generated, it maintained strong predictive performance (PCC = 0.870). The performance difference between BindCraft-only features and embeddings was relatively small, with BindCraft achieving a PCC of 0.810. When the features were combined by appending 14 BindCraft energy terms to the 2560-dimensional embedding input, the performance gain was modest, with a PCC increase of only 0.008. A substantial number of complexes produced zero-valued BindCraft feature vectors and were excluded from the analysis.

### 3.6 Protein split

Protein-level splitting is essential for a fair assessment of generalization. In pairwise affinity prediction datasets, many entries correspond to mutants of the same wild type or to closely related homologs. Random splits therefore place near-duplicate complexes in both training and testing sets, allowing models to memorize scaffold-specific features, reuse shared alignment signals, and interpolate around the wild-type sequence. This leakage can artificially inflate correlation metrics and deflate prediction error.

Protein-level splitting instead groups records by complex-level identifier (PDB ID), assigning each wild-type complex and all its mutants entirely to either the training or testing set. This strategy prevents information leakage from similar embeddings and sequence context. Unlike sequence-identity-based clustering, protein-level splitting does not group complexes by receptor or ligand similarity, because binding affinity depends on the specific interacting partner pair, as the same receptor can exhibit vastly different affinities with different ligands. Although protein-level splits typically yield lower absolute performance metrics and increased fold-to-fold variability, they provide a more realistic evaluation of performance on unseen protein complexes and offer more reliable guidance for downstream design and screening applications.

**Figure 6:**
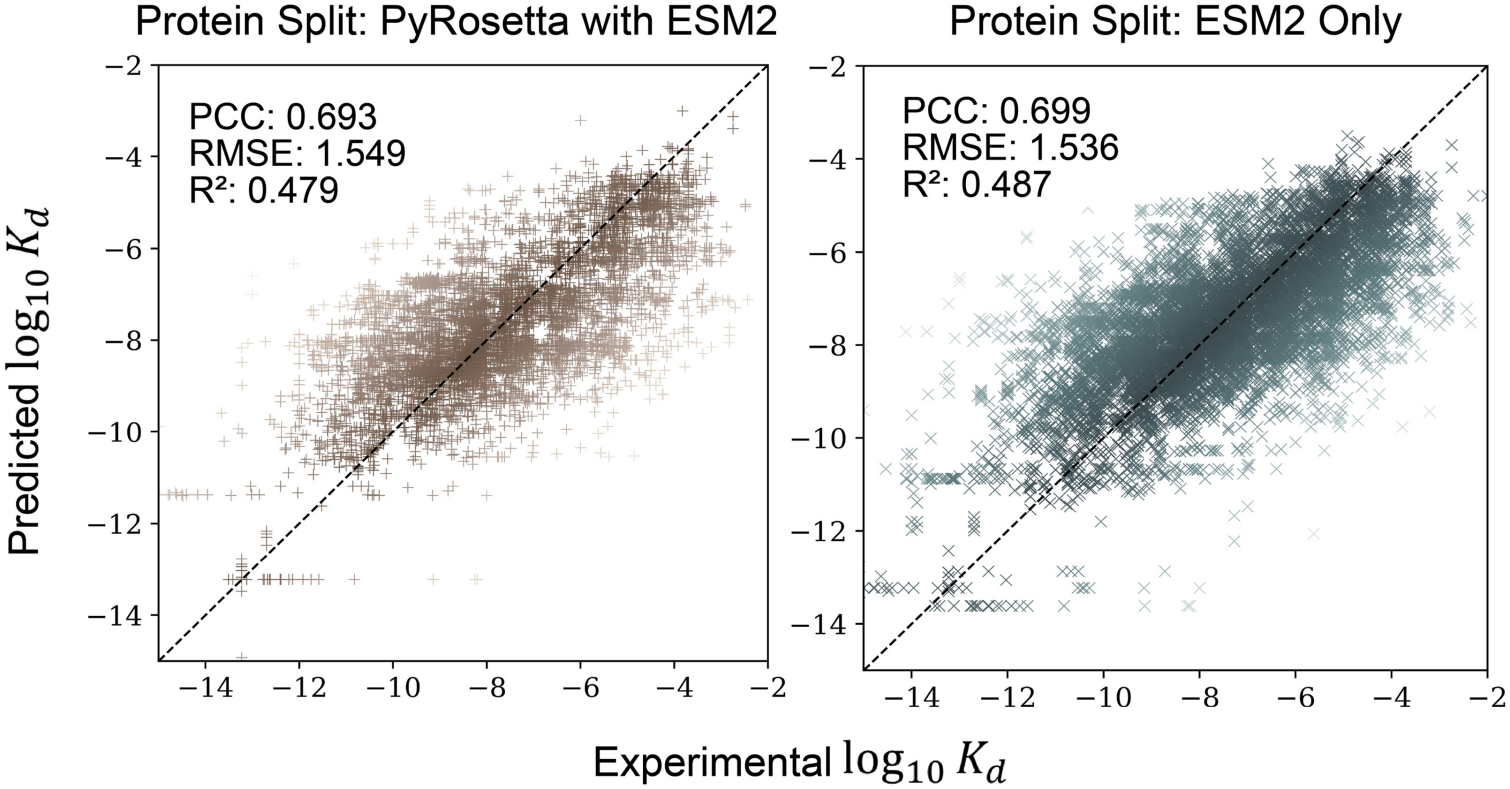
Protein-level split fivefold cross validations. Scatter plots compare predicted versus experimental $\log_{10}K_{d}$ for protein-level five-fold cross-validation, where all variants of a protein are placed entirely in training or testing sets. Both ESM2 alone and the PyRosetta with the ESM2 model exhibit lower performance under protein split than under random split, as expected from a stricter test of generalization. However, their performance remains closely comparable, even though the latter includes structural energy terms.

Figure 6 demonstrates similar performance trends under a protein-level split, indicating that the inclusion of physics-based descriptors provides limited incremental predictive information beyond what is already captured by the embeddings. Under this evaluation framework, incorporating explicit energy terms did not materially improve predictive accuracy. Protein embeddings encode residue identity and contextual sequence dependencies correlated with structural packing and local chemical environments, providing a plausible mechanistic basis for their strong stand-alone performance. From a practical standpoint, the embedding-only model achieved comparable predictive accuracy while reducing computational overhead and feature engineering complexity. These results further suggest that the additional physics-based descriptors may not provide sufficiently orthogonal information relative to the embedding representations under the evaluated conditions. Any incremental benefit from explicit energy features would therefore be more likely to emerge in regimes where evolutionary sequence signals are sparse, chemical environments are atypical, alignment depth is limited, or where evaluation involves distribution shifts that extend beyond those imposed by a protein-level split.

### 3.7 SOTA comparison

This state-of-the-art comparison was conducted using a controlled cross-dataset evaluation to assess model generalization. Both the ESM2-based BindPred model and ProtT-Affinity (Lou 2025) were retrained from scratch on the PDBbind v2020 dataset, and the model performance was evaluated on the same held-out Affinity Benchmark v5.5 test set to ensure a consistent comparison protocol.

The Affinity Benchmark v5.5 dataset contains a substantial proportion of multichain protein complexes with increased structural and interfacial complexity, whereas PDBbind v2020 is enriched in enzyme-inhibitor and antibody-antigen complexes that typically exhibit more localized and structurally constrained interfaces (Guest *et al.* 2021). This train-test mismatch provides a stringent assessment of cross-domain generalization.

As external baselines, ProtT-Affinity represents an end-to-end transformer-based embedding model, whereas PRODIGY (Honorato *et al.* 2021) represents a structure-based empirical scoring approach. PRODIGY was evaluated on the same experimental structures as the embedding-based models using recommended default settings. This design enables comparison across three modeling strategies: embedding-based regression (BindPred), transformer fine-tuning (ProtT-Affinity), and physics-inspired structural scoring (PRODIGY).

**Figure 7:**
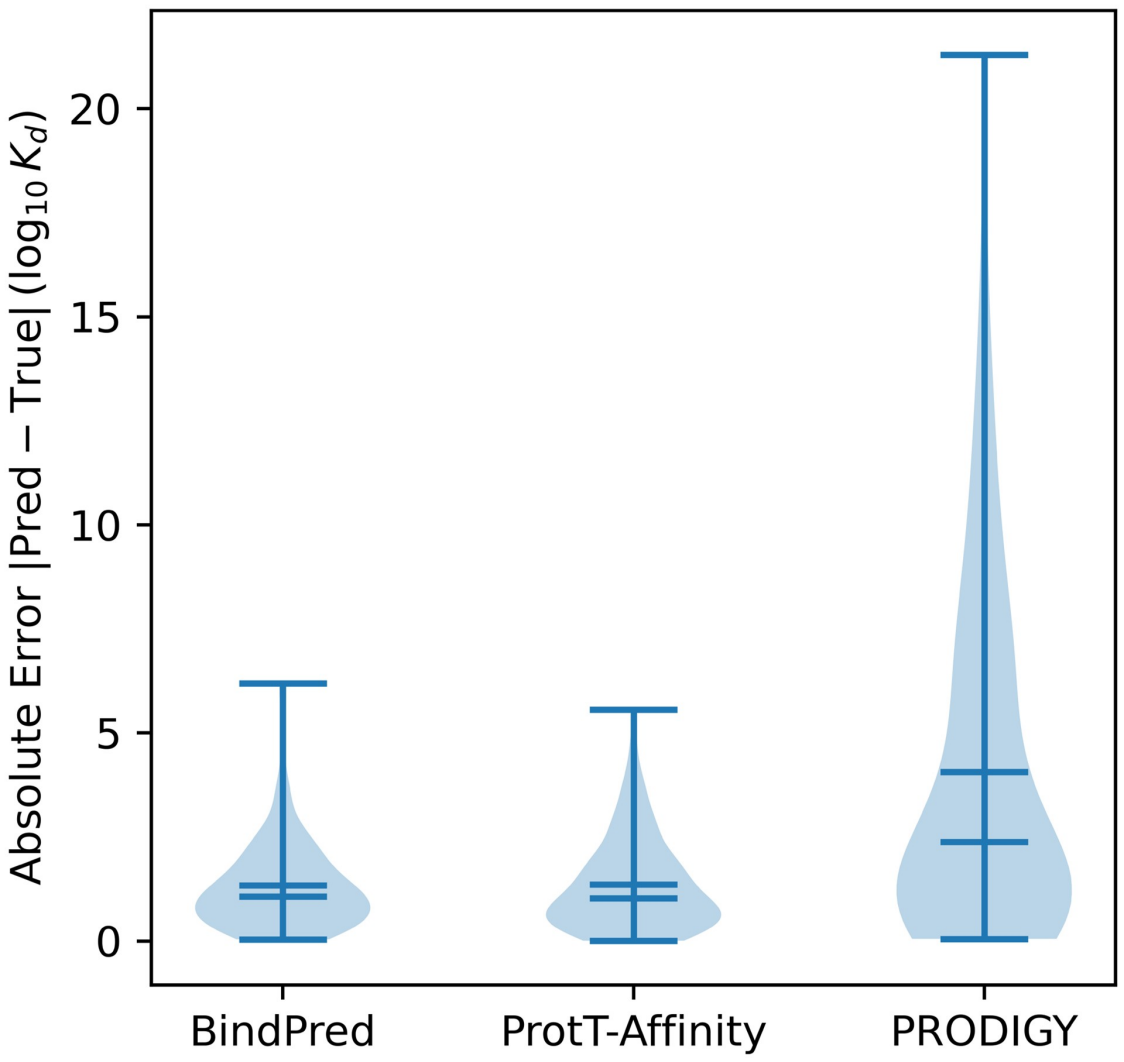
Absolute error distribution across binding-affinity prediction models. Violin plots depict the distribution of absolute prediction errors where the width of each violin represents the density of samples at a given error magnitude, while the internal markers indicate summary statistics of the distribution. BindPred and ProtT-Affinity exhibit relatively compact error distributions, with most predictions concentrated at lower error values and limited spread toward large deviations. In contrast, PRODIGY shows a substantially broader distribution with a pronounced long tail, indicating a higher frequency of large prediction errors and greater variability in performance. Overall, the narrower distributions observed for embedding-based models suggest improved consistency and robustness in multichain complex binding affinities.

As shown in Fig. 7, BindPred and ProtT-Affinity exhibit comparable predictive performance on the Affinity Benchmark v5.5 test set, with similar absolute error distributions. In contrast, PRODIGY displays broader error distribution and increased variance. The reduced performance of PRODIGY on this benchmark is consistent with the known sensitivity of empirical interface descriptor models to multichain structures and increased structural heterogeneity within the test dataset.

### 3.8 Evaluation of complex-level affinity prediction for *de novo* designed sequences

To evaluate whether sequence-based affinity prediction can assist in assessing *de novo* protein designs, BindPred was applied to a set of designed protein complexes from the BindCraft supplementary dataset. These designs from BindCraft were generated using structure-constrained sequence optimization and evaluated within design workflows using Rosetta $\Delta G_{\mathrm{interface}}^{\mathrm{REU}}$, commonly referred to as binder energy. Importantly, Rosetta $\Delta G_{\mathrm{interface}}^{\mathrm{REU}}\;$is an empirical interface energy score optimized for ranking structural models and does not represent an experimentally calibrated measure of absolute binding affinity.

Because binder energy reflects relative interface stability rather than binding affinities, the predicted $\log_{10}K_{d}$values from BindPred were compared against Rosetta $\Delta G_{\mathrm{interface}}^{\mathrm{REU}}\;$to assess ranking concordance between sequence-based and structure-based scoring strategies. A moderate positive correlation was observed between BindPred-derived rankings and Rosetta binder energy rankings (PCC = 0.59, *n* = 212), indicating partial agreement in prioritizing candidate binders while also reflecting differences in the determinants captured by each approach. Whereas BindPred infers affinity from sequence-derived embedding features, Rosetta evaluates structural interactions through empirically weighted physical energy terms.

## 4 Discussion

This work establishes BindPred as a sequence-driven framework for estimating absolute protein–protein binding affinity using protein language model embeddings combined with lightweight regression modeling. The results demonstrate that sequence-derived representations capture substantial biochemical and interaction-relevant information, enabling accurate affinity prediction without explicit structural input. Across multiple validation schemes and heterogeneous interaction datasets, embedding-based models maintain robust performance while preserving computational efficiency.

The limited performance gain observed upon incorporation of explicit structural energy descriptors suggests that evolutionary sequence information encoded in large-scale language model embeddings already reflects many determinants of interfacial energetics. Similar generalization–specialization trade-offs have been reported in enzyme kinetic parameter prediction, where globally trained models leverage heterogeneous datasets yet may sacrifice fine-grained accuracy relative to locally trained, family-specific predictors (Malli *et al.* 2026).

Because BindPred operates exclusively on sequence input, it can be integrated into protein engineering workflows as a rapid post-design screening tool. In particular, it complements sequence generation approaches such as ProteinMPNN (Dauparas *et al.* 2022) and related inverse-folding or generative design frameworks, which optimize structural compatibility and interface stability but do not directly estimate experimentally measurable binding affinity. BindPred therefore provides a sequence-level prioritization mechanism that can rank candidate binders prior to structural refinement or experimental validation, facilitating more efficient multi-stage design pipelines.

The comparable performance of gradient boosting and deep neural networks suggests that feature quality may be more important than architectural choice for binding affinity prediction, as supported by the comparison with PPB-Affinity baseline model and ProtT-Affinity. A simple gradient boosting regressor trained on embeddings matched the accuracy of more elaborate neural networks, consistent with prior studies (Wu *et al.* 2024, Reim *et al.* 2025) that found improvements from ESM2 embeddings were largely insensitive to model architecture. Future work could explore other embedding approaches or fine-tuning strategies for difficult cases where evolutionary information is limited.

## Supplementary Material

btag309_Supplementary_Data

## Data Availability

The pretrained model and inference pipeline are available in a Google Colab notebook: BindPred Colab notebook. The training dataset, code, and model weights are available on the hugging face: https://huggingface.co/hbp5181/BindPred.
